# Predictive models in extracorporeal membrane oxygenation (ECMO): a systematic review

**DOI:** 10.1186/s13643-023-02211-7

**Published:** 2023-03-15

**Authors:** Luca Giordano, Andrea Francavilla, Tomaso Bottio, Andrea Dell’Amore, Dario Gregori, Paolo Navalesi, Giulia Lorenzoni, Ileana Baldi

**Affiliations:** 1 Unit of Biostatistics, Epidemiology and Public Health, Department of Cardiac Thoracic Vascular Sciences and Public Health, University of Padova, Via Loredan 18, 35121 Padova, Italy; 2 ClinOpsHub s.r.l., Via Manfredi Svevo 30 B, 72023 Mesagne, Brindisi, Italy; 3 Thoracic Surgery Unit, Department of Cardiac Thoracic Vascular Sciences and Public Health, University of Padova, Via Loredan 18, 35121 Padova, Italy; 4 Department of Medicine (DIMED), Institute of Anesthesia and Intensive Care, University of Padova, Padova, Italy

**Keywords:** ECMO, Extracorporeal membrane oxygenation, Mortality, Predictive models, Predictive scores

## Abstract

**Purpose:**

Extracorporeal membrane oxygenation (ECMO) has been increasingly used in the last years to provide hemodynamic and respiratory support in critically ill patients. In this scenario, prognostic scores remain essential to choose which patients should initiate ECMO.

This systematic review aims to assess the current landscape and inform subsequent efforts in the development of risk prediction tools for ECMO.

**Methods:**

PubMed, CINAHL, Embase, MEDLINE and Scopus were consulted. Articles between Jan 2011 and Feb 2022, including adults undergoing ECMO reporting a newly developed and validated predictive model for mortality, were included. Studies based on animal models, systematic reviews, case reports and conference abstracts were excluded. Data extraction aimed to capture study characteristics, risk model characteristics and model performance. The risk of bias was evaluated through the prediction model risk-of-bias assessment tool (PROBAST). The protocol has been registered in Open Science Framework (https://osf.io/fevw5).

**Results:**

Twenty-six prognostic scores for in-hospital mortality were identified, with a study size ranging from 60 to 4557 patients. The most common candidate variables were age, lactate concentration, creatinine concentration, bilirubin concentration and days in mechanical ventilation prior to ECMO. Five out of 16 venous-arterial (VA)-ECMO scores and 3 out of 9 veno-venous (VV)-ECMO scores had been validated externally. Additionally, one score was developed for both VA and VV populations. No score was judged at low risk of bias.

**Conclusion:**

Most models have not been validated externally and apply after ECMO initiation; thus, some uncertainty whether ECMO should be initiated still remains. It has yet to be determined whether and to what extent a new methodological perspective may enhance the performance of predictive models for ECMO, with the ultimate goal to implement a model that positively influences patient outcomes.

**Supplementary Information:**

The online version contains supplementary material available at 10.1186/s13643-023-02211-7.

## Take-home message

This systematic review identified 26 predictive prognostic models for ECMO developed in the last 10 years.

Most models have not been validated externally and uncertainty if ECMO should be initiated or not still remains. It has yet to be determined whether and to what extent a new methodological perspective may enhance the performance of predictive models for ECMO.

## Introduction

Extracorporeal membrane oxygenation (ECMO) is an advanced technique that involves oxygenation of blood outside the body and supports selected patients with severe respiratory or cardiac failure [1], which can provide short-term mechanical support for the heart, lungs or both. Despite having been used for the first time clinically in the 1970s [2], the increased use of ECMO among seriously ill adult patients is a recent phenomenon [3]. Over the last decades, ECMO has remarkably progressed and has become a widely used rescue therapy for severe respiratory or cardiac disease refractory to other procedures [4]. Similarly, indications of ECMO in adults have extended beyond severe acute respiratory failure and heart failure [5] to include extracorporeal cardiopulmonary resuscitation (ECPR) [6] and as a bridge to lung transplantation [7]. As such, ECMO is considered a lifesaving procedure.

Two major ECMO modalities exist, veno-venous ECMO (VV-ECMO) and veno-arterial ECMO (VA-ECMO) [8, 9]. The former provides pulmonary support when gas exchange is severely compromised after any potentially reversible acute respiratory failure: common scenarios include acute respiratory distress syndrome (ARDS) and pneumonia [9]. The latter is a form of both pulmonary and circulatory support reserved for acute cardiorespiratory failure; it is commonly used in cardiac arrest and in cardiogenic shock (CS) [10].

Despite being a lifesaving therapy, several studies have reported different mortality rates depending on indication and modality [11], ranging from 76% in one cohort undergoing ECMO and dialysis [12] to 37% in a mixed veno-venous (VV)/veno-arterial (VA) ECMO group [13]. Between 1989 and 2014, the ELSO registry reported mortality rates for respiratory and cardiac ECMOs at 57% and 41%, respectively [14]. Moreover, a number of complications can occur, of which any can inflict severe morbidity. In addition, the procedure is highly invasive with severe complications and is time- and resource-consuming [15–18].

Moreover, the possibility to initiate ECMO largely relies on ECMO machines availability, and a trained, multidisciplinary team composed of critical care physicians, cardiac surgeons, perfusionists and expert nurses, among others. Recently, there has been a shortage of ECMO machines [19] and specialized personnel [20]. Due to these factors, the effective possibility of offering ECMO to the patients often has some limitations and varies from country to country.

Thus, a predictive model able to identify the patients potentially benefitting from ECMO is of utmost importance.

Several predictive models exist. Non-specific ECMO scores were initially developed to assist critically ill patients and were tested for predictive accuracy in a population undergoing ECMO. In this context, some notable examples are the Sequential Organ Failure Assessment (SOFA) [21] score and the Acute Physiology and Chronic Health Disease Classification System II (APACHE II) [22]. Contradictory results have been shown for their discrimination and calibration. Hilder et al. [23] reported that the SOFA score showed better discrimination than some ECMO-specific scores in VV-ECMO population. On the other hand, Fisser et al. [24] showed that these generic intensive care unit (ICU) scores were better than specific scores in VA-ECMO population but worse in VV-ECMO population.

Specific ECMO models have been developed in the past years to overtake these limits. While predicting better survival compared to general risk scores used in the ICU [22], there is a shared perception that specific ECMO models are unsatisfactory in supporting the decision-making process. Given these considerations, a close examination of the recently developed ECMO scores, underlining their statistical properties, strength and limitations, could be a matter of relevance.

This should be the starting point for any future research on ECMO scoring systems either when the aim is to identify and update promising scores or to build a score that could be used before the ECMO initiation, adding information to the clinical judgement and helping to decide if a patient should receive ECMO or not.

We conducted a systematic review to provide a critical appraisal of existing prognostic scores in the adult population undergoing ECMO therapy and their evidence supporting their utilization in clinical practice.

## Methods

This review was conducted with adherence to the Preferred Reporting Items for Systematic Review and Meta-Analyses (PRISMA) guidelines [25]. The protocol has been previously published with Open Science Framework (https://osf.io/fevw5).

The PICO framework was defined as follows: the population is represented by adult population in ICU; the intervention is the initiation of ECMO therapy; a comparator is not possible to define, as this study focuses on exposed to ECMO; and the outcome is the mortality.

### Search

Mortality following ECMO initiation in adult population was the objective of our investigation. Our systematic review is for the most part descriptive and involves no statistical analysis. We wanted to shed some light on an unexplored landscape. Over the years, there have been tireless improvements in all aspects of the use of ECMO: technology, patient selection and management and a broader understanding of this complex therapy. Thus, we limited the review to the last 10 years to exclude potentially outdated scores. Five electronic databases were queried: PubMed, MEDLINE, CINAHL, Embase and Scopus.

We created the following search string for PubMed database: ("extracorporeal membrane oxygenation"[MeSH Terms] OR ("extracorporeal"[All Fields] AND "membrane"[All Fields] AND "oxygenation"[All Fields]) OR "extracorporeal membrane oxygenation"[All Fields] OR "ecmo"[All Fields]).

To better adapt the search string to the other databases, we translated the original string, thanks to Polyglot Search Tool [26].

A complete list of strings created by Polyglot Search Tool is available in supplementary materials (Table S1).

Studies published between 1 Jan 2011 and 27 Feb 2022 were eligible for inclusion. In this phase, no restrictions have been applied on the language or type of publication. The complete citation list obtained in the previous stage was saved for future exploration. To do this, all the files derived from databases interrogation were imported into Zotero citation manager [27] and then exported as a unique RIS file.

The deduplication phase was performed with the help of the Institute for Evidence-Based Healthcare (IEBH) offline de-duplicator [28]. Once the automatic deduplicator phase finished, the file containing all the citations deduplicated was uploaded in Rayyan [29], an intelligent research tool used for the title and abstract review phase.

Two authors, A. F. and L. G., were involved in reviewing the titles and abstracts of all the citations. The third author (I. B.) was in charge of resolving any conflict between the two original reviewers.

### Inclusion and exclusion criteria

Studies reporting the development of a predictive model for death in critically ill adult patients undergoing VA- or VV-ECMO were selected for further exploration. Death following the initiation of ECMO was the outcome of interest. English or Italian language was accepted. Lastly, we chose to include studies worldwide, with no geographical restrictions. Exclusion criteria were represented by the specific usage of ECMO: in order to focus on modalities of ECMO that give full support to the patients, ECMO as a bridge and ECMO used to deliver CO2 removal only (ECCO2R) were excluded. The studies based on single predictors were excluded as well. Additional exclusion criteria were represented by the following: studies that did not report a newly developed model but used a previously validated model and studies based on animal models, systematic reviews, case reports and conference abstracts.

### Data extraction

For each included study, the following information was extracted: title, first author, model name, model type, study type, geographic area, ECMO type, number of patients included, endpoint, setting, candidate variable considered for model derivation, final model variables and model discrimination, calibration and validation as reported by the authors. Finally, the area under the ROC curve (AUC-ROC) — for internal and external validation if available — as a measure of the model discriminatory ability has been captured.

### PROBAST

Each identified model was independently evaluated by two authors (A. F. and L. G.) for risk of bias (ROB) through the Prediction model Risk Of Bias Assessment Tool (PROBAST), according to Moons et al. [30]. A third author (I. B.) oversaw resolving any conflict between the two original raters. Each model was assessed for applicability concerns and ROB, based on four domains. If a domain of interest was evaluated “no [N]” or “probably no [PN]”, there was supposed to be a concern for applicability or potential for bias within that domain. If the review questions were satisfied, concern regarding applicability was rated overall “low”.

A publication is needed to score “low ROB” in each of the four domains for an overall judgment of “low ROB”.

If a single domain was marked as “high ROB”, overall “low ROB” could theoretically still be stated, but specific reasons should be provided.

## Results

The bibliographic research generated a total of 101,898 references. After deduplication phase, 51,036 unique articles were suitable for title and abstract screening.

After title and abstract screening, 174 references remained for full-text screening.

**Fig. 1:**
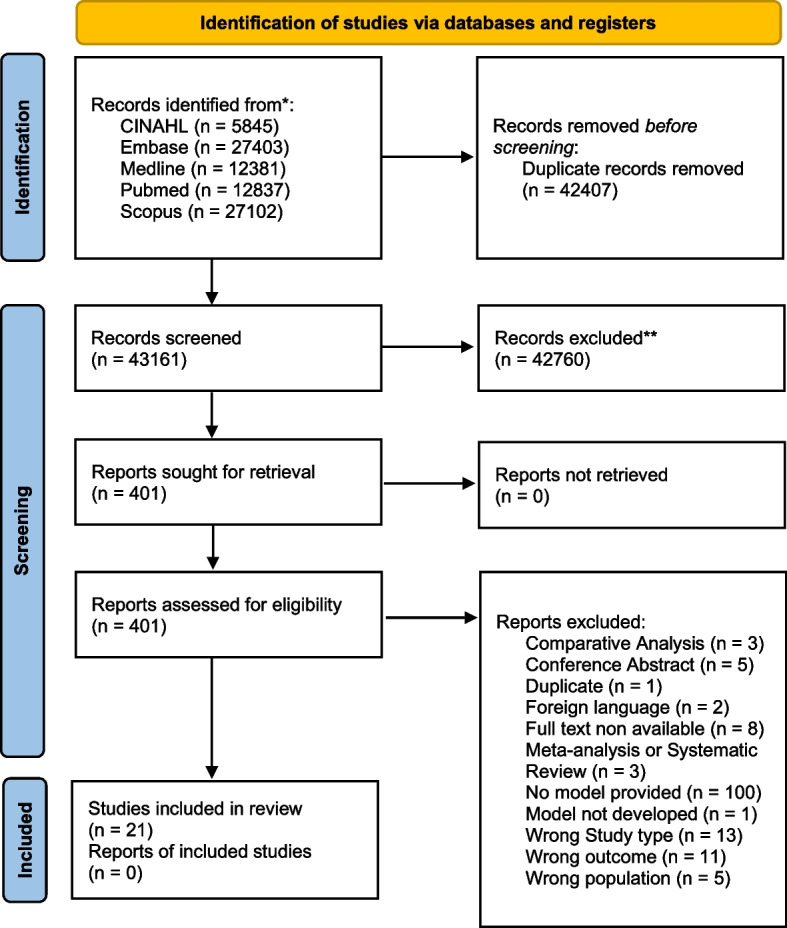
PRISMA flowchart

At the end of full-text review, a total of 24 references fully met our inclusion criteria and were considered eligible for data extraction. Further information can be found in the PRISMA flowchart in Fig. 1.

These 24 references reported 26 models with in-hospital mortality as the outcome (two of these 24 references built two different models). The studies are heterogeneous with respect to sample size and patients’ populations (Table S2).

Nine models were developed in Europe, eight in Asia, seven in America and two in Oceania. These models included a minimum of three up to a maximum of 18 variables: the total number of variables considered in the final model often contributes (as it should) to determine the relative weight of each variable (Table S2).

Interestingly, 22 models out of 26 (85%) relied on generalized linear model (GLM) to build the model; for the remaining four, one relied on generalized estimating equation (GEE), one on Cox proportional hazards and one on deep neural networks (DNN) and the last one on GLMNet.

**Fig. 2:**
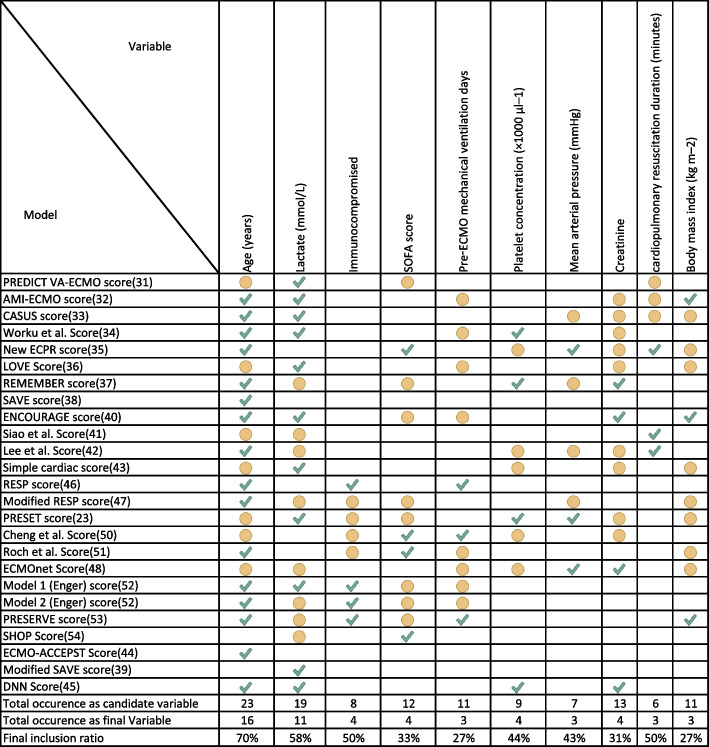
Most represented variables in the models (appearing in at least 2 models) and indication if present only as candidate variables (yellow dot) or if retained in the final model (green check)

The variables mainly involved are, in order of frequency, as follows: age and lactate blood concentration, contained respectively in 18 and in 11 models (Fig. 2).

We found two categories of ECMO models, reflecting two different ECMO modalities: models for VA-ECMO and models for VV-ECMO (Table 1). For VA-ECMO scores, we found the subsequently 16 scores: the PREDICT VA-ECMO [31], the AMI-ECMO [32], the CASUS [33], the Worku et al. score [34], the New ECPR [35], the LOVE [36], the REMEMBER [37], the SAVE [38], the modified SAVE [39], the ENCOURAGE [40], Siao et al. score [41], Lee et al. score [42], the simple cardiac score [43], the ECMO-ACCEPTS score [44] and the deep neural networks (DNN) score [45]. Of these, only 3 have been validated externally (3/14, 21%): the SAVE [38] and two different models of the PREDICT VA-ECMO [31] score, which reported an AUC-ROC for external validation of 0.9 (95% *CI*: 0.85–0.95) and 0.72 (95% *CI*: 0.65–0.8), respectively.

**Table 1:** ECMO-specific scores and their discrimination

				**Internal validation**			**External**
***Model name***	***Type of ECMO***	**Indication**	***Study design***	***Type of model***	***Variable selection***	**AUC (95% *CI*)**	**AUC (95% *CI*)**
**6-h PREDICT VA-ECMO score** [31]	VA	Cardiogenic shock	Retrospective, single centre	GLMNet	Elastic net	0.82 (0.76–0.88) Cross-validation	0.72 (0.65–0.78)
**12-h PREDICT VA-ECMO score** [31]	VA	Cardiogenic shock	Retrospective, single centre	GLMNet	Elastic net	0.84 (0.78–0.9) Cross-validation	0.73 (0.65–0.82)
**AMI-ECMO score** [32]	VA	Acute myocardial infarction	Retrospective, single centre	GLM (logistic)	Two steps: based on univariable *p*-values; backward stepwise	0.88 (0.82–0.94) LOO cross-validation	-
**CASUS score** [33]	VA	Post-cardiotomy; circulatory failure	Retrospective, single centre	GLM (logistic)	-	0.68 (0.57.0.77)	-
**Worku et al. score** [34]	VA	Cardiogenic shock	Retrospective, single centre	GLM (logistic)	Based on univariable *p*-values	-	-
**New ECPR score** [35]	VA	Cardiac arrest	Retrospective, single centre	GLM (logistic)	Two steps: based on univariable and multiple regression *p*-value	0.86 (0.77–0.94) Bootstrap	-
**12-month LOVE score** [36]**	VA	ECMO for every condition	Retrospective, single centre	GLM (logistic)	Combined logistic and Cox	0.75 (0.62–0.88)	-
**REMEMBER score** [37]	VA	Cardiogenic shock following coronary artery bypass grafting	Retrospective, single centre	GLM (logistic)	Two steps: based on univariable *p*-values; backward stepwise	0.85 (0.79–0.91) Bootstrap	-
**SAVE score** [38]	VA	Refractory cardiogenic shock	Retrospective based on ELSO registry	GLM (logistic)	Two steps: based on univariable and multiple regression *p*-value	0.68 (0.64–0.71) Bootstrap	0.90 (0.85–0.95)
**Modified SAVE score** [39]	VA	ECMO for every condition	Retrospective, single centre	GLM (logistic)	Two steps: based on univariable *p*-values; forward stepwise	0.84	
**ENCOURAGE score** [40]	VA	AMI-related refractory cardiogenic shock patients	Retrospective, two centres	GLM (logistic)	Two steps: based on univariable *p*-values; backward stepwise	0.84 (0.77–0.91) Bootstrap	-
**Siao et al. score** [41]	VA	Cardiac arrest	Retrospective, single centre	GLM (logistic)	Based on univariable *p*-values	- Bootstrap	-
**Lee et al. score** [42]	VA	Cardiac arrest	Retrospective, single centre	GLM (logistic)	Two steps: based on univariable and multiple regression *p*-value	0.86 (0.80–0.93) Bootstrap	-
**Simple cardiac score** [43]	VA	Any form	Retrospective, single centre	GLM (logistic)	Two steps: based on univariable and multiple regression *p*-value	0.77 (-)	-
**ECMO-ACCEPTS score** [44]	VA	Any form excluding respiratory leading cause	Retrospective, based on administrative data	Cox proportional hazards	Two steps: based on univariable and multiple PH	0.64 (0.63–0.66)	-
**DNN score** [45]	VA	Any form	Retrospective, single centre	Deep neural network	Learning vector quantization	0.92 3 × 10-fold cross-validation	-
**SHOP score** [46]	VA/VV	Any form	Retrospective, single centre	GLM (logistic)	Two steps: based on univariable and multiple regression *p*-value	0.87 (0.79–0.96)*	-
**RESP score** [47]	VV	Respiratory failure	Retrospective based on ELSO registry	GLM (logistic)	Based on multiple regression *p*-value	0.74 (0.72–0.76) Bootstrap	0.92 (0.89–0.97)
**Modified RESP score** [48]	VV	Respiratory failure	Retrospective, single centre	GLM (logistic)	Two steps: based on univariable *p*-values; forward stepwise	0.71 (0.63–0.78)	-
**PRESET score** [23]	VV	Respiratory failure	Retrospective, single centre	GLM (logistic)	Two steps: based on univariable *p*-values; backward stepwise	0.85 (0.76–0.93)	0.7 (0.56–0.83)
**Cheng et al. score** [49]	VV	Respiratory failure	Retrospective, single centre	GLM (logistic)	Two steps: based on univariable *p*-values; backward stepwise	0.76 (0.67–0.85)	-
**Roch et al. score** [50]	VV	ARDS	Retrospective, single centre	GLM (logistic)	Two steps: based on univariable and multiple regression *p*-value	0.8 (0.71–0.89)	-
**ECMOnet score** [51]	VV	ARDS due to H1N1 influenza	Prospective multi-centre	GEE	Two steps: based on univariable and multiple regression *p*-value	0.86 (0.75–0.96)	0.69 (0.56–0.83)
**Model 1 (Enger) score** [52]	VV	Respiratory failure	Retrospective, single centre	GLM (logistic)	Clinical choice	0.75 (0.69–0.8) Bootstrap	-
**Model 2 (Enger) score** [52]	VV	Respiratory failure	Retrospective, single centre	GLM (logistic)	Clinical choice	0.79 (0.73–0.84) Bootstrap	-
**PRESERVE score** [53]	VV	ARDS	Retrospective multi-centre	GLM (logistic)	Two steps: based on univariable *p*-values; backward stepwise	0.89 (0.83–0.94)	-

For VV-ECMO, 9 scores have been individuated: the RESP score [47], the modified RESP score [48], the PRESET score [23], the ECMOnet [51], Cheng et al. score [49], Roch et al. score [50], model 1 and model 2 by Enger et al. [52] and the PRESERVE score [53]. Out of 9 scores, three have been validated externally (4/10, 40%): the RESP score [47] (AUC-ROC = 0.92 [95% *CI* 0.89–0.97], the PRESET score [23] (AUC-ROC = 0.7, 95% *CI*: 0.56–0.84) and the ECMOnet score [51] (AUC-ROC = 0.69, 95% *CI*: 0.56–0.82).

Additionally, the SHOP score [46] was developed lastly and has been tested on both VA and VV populations. For both VA and VV, it has very promising AUC-ROC (aggregate: 0.87 [0.79–0.96]); however, it has yet to be validated externally.

### PROBAST

None of the models out of 26 (0%) was classified as overall low ROB. While in “participants”, “predictors” and “outcome” domains, the models generally do well, with an overall ROB classified as low in 13/26 scores (50%) and as high the remaining 50%; the last domain — reserved to the analysis methods — is the one where the totality of the scores fails.

**Fig. 3:**
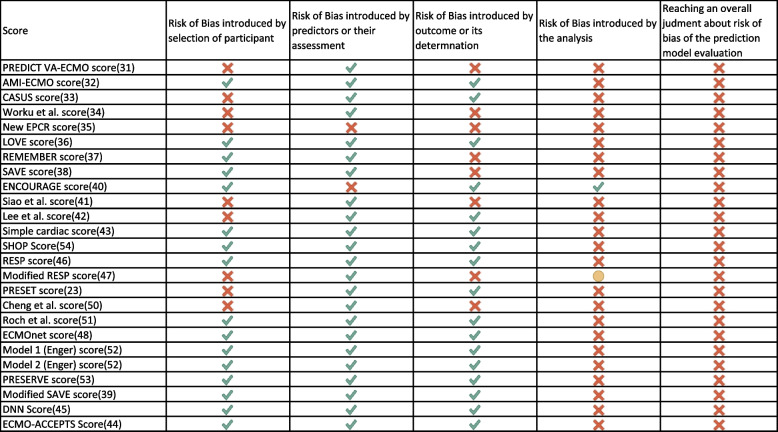
List of ECMO-specific scores and their risk of bias (ROB). The red cross mark indicates high ROB, the yellow dot indicates unclear risk, while the green check indicates low ROB

In particular, the validation process does not take into account both the discrimination or the calibration of the reported model at the same time. A summary of ROB can be displayed in Fig. 3.

## Discussion

ECMO use continues to rise rapidly in adults [44]. Since ECMO therapy requires highly specialized staff and equipment, it is crucial to select and monitor patients stratified by outcome prediction for this economically costly and risky therapy. Equally, basing medical decisions on mis-calibrated predictions can be harmful.

This review identified 26 different predictive prognostic models for mortality developed in the last decade.

Together, age and lactatemia are by far the most represented variables. The number of days in mechanical ventilation before the start of the ECMO was used in several scores. Additional variables were the renal functionality parameters (creatinine, bicarbonate, pH), the cardiorespiratory parameters (minute ventilation, mean arterial pressure, cardiomyopathy, FiO2), Glasgow Coma Scale (GCS), immunity system, blood pressure and body temperature.

Multivariable logistic regression was the underlying statistical model for the most part. Candidate predictors were entered into the model, and a subset was finally retained after undergoing a variable selection process.

The major lacking area that arose from analysing the prediction models included reporting on calibration methods.

### Advantages and disadvantages of validated scoring systems

PREDICT VA-ECMO [31] shows limited discriminatory ability. The SAVE score [38] achieves better performance, justifying its widespread usage. Nevertheless, as painstakingly pointed out by the authors of the SAVE score [38], their information — yet precious — only offers a partial answer to the patient selection problem. In fact, they consider patients’ characteristics only after VA-ECMO initiation, not establishing if VA-ECMO should be initiated or not based on patients’ baseline characteristics prior to ECMO initiation. Additionally, complete data was available for only 23% (876/3846) in ECMO cohort.

The PRESET exhibits low discrimination in the validation set. Additionally, it presents a potential bias in patient selection and population outcome, with higher mortality reported in internal and external cohorts than the mortality in analogous clinical settings [54]. In advance, the external validation was made on a single medical centre (59 patients). The ECMOnet score [51], while presenting a robust selection of patients and showing good discrimination in its validation set, has a relatively small sample and should be further validated in bigger validation sets, as the authors [51] and other centres [55] conclude. In advance, the ECMOnet score focuses on H1N1 patients, so it could be helpful on this cohort of patients.

The RESP score [47] has, together with the PRESERVE score [53], which had only internal validation in his development article, shown modest performance when subsequent studies tried to validate them in an external cohort [56].

### Challenges of building an ECMO scoring system

It is imperative to remind certain facts regarding predictive scores. First, their development should follow the TRIPOD checklist (www.tripod-statement.org). Second, a prediction model should be developed on a multicentric prospective cohort with a clearly defined outcome. Third, the statistical analysis should rely on a sensitivity analysis of different models’ specifications and not necessarily limited to traditional regression models. Fourth, it is crucial to use risk factors that are easy to measure in a standardized way across populations if we intend for the model to be applicable in different settings. This brings up the dilemma of specialization versus generalizability. Fifth, when choosing a predictive scoring system, it is mandatory to consider its prognostic capability and validation type. A score that has not been validated externally is susceptible to several issues — overfitting being the most common — and should be interpreted with more caution. In addition, feasibility, ease of use and cost should also be taken into consideration. Finally, the interpretation of a predictive score should be cautiously reported.

We identified weaknesses in the type of outcome and follow-up time, in the statistical model and in the calibration process. All the considered models exclusively analyse in-hospital mortality, neglecting the importance of other fundamental outcomes such as medium/long-term mortality or the prevalence of major outcomes in survivors.

The scores found relied on logistic regression for the most part. While logistic regression can provide very important pieces of information and can perform very well, more advanced models — guided by computational methods (e.g. machine learning) — could potentially offer promising results [57, 58].

The issue of calibration was partially neglected even if it is especially important when the aim is to support decision-making [59]. Calibration is the accuracy of risk estimates, relating to the agreement between the estimated and observed number of events. Irrespective of how well the models can discriminate between ECMO patients that die versus those that do not in a given time frame, it is clear that strong over- or underestimation of mortality could make a score clinically unacceptable. Indeed, if a physician is supported in her decision by a not well-calibrated score, this could lead to under- or overtreatment. For example, by relying on a score overestimating the risk, a physician could decide not to treat a patient above a given threshold even if that patient would respond well to the treatment because his/her true underlying risk is lower. For these reasons, it is mandatory to calibrate the scores once built, even when the discrimination is strong.

Most of the predictive models about ECMO focus on discrimination but do not consider a proper calibration.

Time has come to refrain from developing new models and redirect that energy towards modelling enhancement. The small sample size is a general problem in many single-centre ECMO studies. It poses a further constraint to the number of variables retained in the final risk models and, in turn, their generalizability. One question which remains still unanswered is whether and to what extent a new methodological perspective may increase discriminatory ability and calibration of predictive models for ECMO, with the ultimate goal to clinically implement a model that has a positive influence on patient outcomes.

### Strengths and limitations

In summary, all existing predictive scoring systems still have some limitations [60, 61]. Anyway, even good risk scores will become obsolete as ECMO technology changes and clinical knowledge increases, and there is a permanent need for updating. Generating a risk model requires careful thinking and a combination of statistical expertise and sound domain knowledge. Modelling and prediction, along with future tools and processes, should borrow from existing ones to build new ones for future readiness. The strength of the present study is to assess the state of the art through a systematic and broad search strategy across major databases in order to identify all studies developing a scoring system for ECMO. To our knowledge, this is the first systematic review to explore this topic.

Some weaknesses of this review must be acknowledged. First, this systematic review tries to explore a very thorny topic; the field itself is very heterogeneous, and a metanalysis has not been conducted. Second, the use of automatic tools for conducting a systematic review can be tricky. Although these tools are gaining popularity [62], they could potentially introduce biases. For example, Rayyan screening system [29] ranks articles by the likelihood of relevance, rather than simply providing definitive, dichotomized classifications. However, even low-ranking articles have some nonzero probability of being relevant, and there remains the possibility of missing a relevant article. Second, a pooling of study-level performance measures was judged unsuitable for this systematic review. Furthermore, another limitation factor could be the selection of research databases. We focused our attention on PubMed, Scopus, Embase, CINAHL and MEDLINE since those are the more relevant databases for clinical research. For this reason, we decided not to include other databases like Google Scholar, IEEE Xplore and Web of Science.

## Conclusions

Notwithstanding a few notable examples, most risk models for ECMO represent an interesting experiment that can be enhanced with the help of the modern computational instruments. Most models have not been validated externally or are built with some weakness, preventing them from being used in clinical practice. In addition, most of these models were created retrospectively on patients already undergoing ECMO. Such an approach can be useful to assess whether to continue to treat the patient with this invasive therapy and thus reduce the risk of therapeutic overkill. It would, however, be interesting to also create and validate a model that can discriminate against patients before they are subjected to ECMO.

## Supplementary Information

**Additional file 1: Table S1.** Search string created by Polyglot Research tool. **Table S2.** Details of ECMO scores.

## Data Availability

Datasets generated during the current study are available from the corresponding author.

## References

[1] Pujara D, Sandoval E, Simpson L, Mallidi HR, Singh SK (2015). The state of the art in extracorporeal membrane oxygenation. Semin Thorac Cardiovasc Surg.

[2] Hill JD, O’Brien TG, Murray JJ, Dontigny L, Bramson ML, Osborn JJ, Gerbode F (1972). Prolonged extracorporeal oxygenation for acute post-traumatic respiratory failure (shock-lung syndrome): use of the Bramson membrane lung. N Engl J Med.

[3] Sauer CM, Yuh DD, Bonde P (2015). Extracorporeal membrane oxygenation use has increased by 433% in adults in the United States from 2006 to 2011. ASAIO Journal.

[4] Extra corporeal membrane oxygenation (ECMO) review of a lifesaving technology. https://www.ncbi.nlm.nih.gov/pmc/articles/PMC4522501/ [Accessed 26 Aug 2021] 10.3978/j.issn.2072-1439.2015.07.17 PMC4522501 26380745

[5] Shekar K, Mullany DV, Thomson B, Ziegenfuss M, Platts DG, Fraser JF (2014). Extracorporeal life support devices and strategies for management of acute cardiorespiratory failure in adult patients: a comprehensive review. Crit Care.

[6] Mosier JM, Kelsey M, Raz Y, Gunnerson KJ, Meyer R, Hypes CD, Malo J, Whitmore SP, Spaite DW (2015). Extracorporeal membrane oxygenation (ECMO) for critically ill adults in the emergency department: history, current applications, and future directions. Crit Care.

[7] Schechter MA, Ganapathi AM, Englum BR, Speicher PJ, Daneshmand MA, Davis RD, Hartwig MG (2016). Spontaneously breathing extracorporeal membrane oxygenation support provides the optimal bridge to lung transplantation. Transplantation.

[8] Rao P, Khalpey Z, Smith R, Burkhoff D, Kociol RD (2018). Venoarterial extracorporeal membrane oxygenation for cardiogenic shock and cardiac arrest. Circ Heart Fai.

[9] Keebler ME, Haddad EV, Choi CW, McGrane S, Zalawadiya S, Schlendorf KH, Brinkley DM, Danter MR, Wigger M, Menachem JN (2018). Venoarterial extracorporeal membrane oxygenation in cardiogenic shock. JACC Heart Fail.

[10] Tsangaris A, Alexy T, Kalra R, Kosmopoulos M, Elliott A, Bartos JA, Yannopoulos D. Overview of veno-arterial extracorporeal membrane oxygenation (VA-ECMO) support for the management of cardiogenic shock. Front Cardiovasc Med. 2021;8: 10.3389/fcvm.2021.686558 [Accessed 13 Dec 2022] 10.3389/fcvm.2021.686558 PMC8292640 34307500

[11] Gray BW, Haft JW, Hirsch JC, Annich GM, Hirschl RB, Bartlett RH (2015). Extracorporeal life support: experience with 2,000 patients. ASAIO Journal.

[12] Tsai C-W, Lin Y-F, Wu V-C, Chu T-S, Chen Y-M, Hu F-C, Wu K-D, Ko W-J (2008). SAPS 3 at dialysis commencement is predictive of hospital mortality in patients supported by extracorporeal membrane oxygenation and acute dialysis. Eur J Cardio Thorac Surg.

[13] Aubron C, Cheng AC, Pilcher D, Leong T, Magrin G, Cooper DJ, Scheinkestel C, Pellegrino V (2013). Factors associated with outcomes of patients on extracorporeal membrane oxygenation support: a 5-year cohort study. Crit Care.

[14] Clark JB, Wang S, Palanzo DA, Wise R, Baer LD, Brehm C, Ündar A (2015). Current techniques and outcomes in extracorporeal life support: invited editorial. Artificial Organs.

[15] El Sibai R, Bachir R, El Sayed M (2018). ECMO use and mortality in adult patients with cardiogenic shock: a retrospective observational study in U.S. hospitals. BMC Emerg Med.

[16] Harvey MJ, Gaies MG, Prosser LA (2015). U.S. and international in-hospital costs of extracorporeal membrane oxygenation: a systematic review. Appl Health Econ Health Policy.

[17] Shah AG, Peahota M, Thoma BN, Kraft WK (2017). Medication complications in extracorporeal membrane oxygenation. Crit Care Clin.

[18] Nasr DM, Rabinstein AA (2015). Neurologic complications of extracorporeal membrane oxygenation. J Clin Neurol.

[19] Farmer B. Even with risky survival rate, shortages of ECMO machines cost lives, study finds. *NPR* (2022) https://www.npr.org/sections/health-shots/2022/03/28/1086212281/ecmo-machine-shortage [Accessed 22 Dec 2022]

[20] Critical shortage of cardiothoracic surgeons anticipated by 2035: researchers urge steps be taken now to train more surgeons to meet burgeoning future needs. *ScienceDaily* https://www.sciencedaily.com/releases/2016/05/160517120520.htm [Accessed 22 Dec 2022]

[21] Magoon R, Shri I, Kohli JK, Kashav R (2020). SOFA scoring in VA-ECMO: plenty to ponder!. J Cardiothorac Vasc Anesth.

[22] Ng WT, Ling L, Joynt GM, Chan KM (2019). An audit of mortality by using ECMO specific scores and APACHE II scoring system in patients receiving extracorporeal membrane oxygenation in a tertiary intensive care unit in Hong Kong. J Thorac Dis.

[23] Hilder M, Herbstreit F, Adamzik M, Beiderlinden M, Bürschen M, Peters J, Frey UH (2017). Comparison of mortality prediction models in acute respiratory distress syndrome undergoing extracorporeal membrane oxygenation and development of a novel prediction score: the PREdiction of Survival on ECMO Therapy-Score (PRESET-Score). Crit Care.

[24] Fisser C, Rincon-Gutierrez LA, Enger TB, Taccone FS, Broman LM, Belliato M, Nobile L, Pappalardo F, Malfertheiner MV (2021). Validation of prognostic scores in extracorporeal life support: a multi-centric retrospective study. Membranes (Basel).

[25] Page MJ, Moher D, Bossuyt PM, Boutron I, Hoffmann TC, Mulrow CD, Shamseer L, Tetzlaff JM, Akl EA, Brennan SE (2021). PRISMA 2020 explanation and elaboration: updated guidance and exemplars for reporting systematic reviews. BMJ.

[26] Clark JM, Sanders S, Carter M, Honeyman D, Cleo G, Auld Y, Booth D, Condron P, Dalais C, Bateup S (2020). Improving the translation of search strategies using the polyglot search translator: a randomized controlled trial. JMLA.

[27] Mueen Ahmed K, Al Dhubaib B (2011). Zotero: a bibliographic assistant to researcher. J Pharmacol Pharmacother.

[28] Rathbone J, Carter M, Hoffmann T, Glasziou P (2015). Better duplicate detection for systematic reviewers: evaluation of systematic review assistant-deduplication module. Syst Rev.

[29] Ouzzani M, Hammady H, Fedorowicz Z, Elmagarmid A (2016). Rayyan—a web and mobile app for systematic reviews. Syst Rev.

[30] Moons KGM, Wolff RF, Riley RD, Whiting PF, Westwood M, Collins GS, Reitsma JB, Kleijnen J, Mallett S (2019). PROBAST: a tool to assess risk of bias and applicability of prediction model studies: explanation and elaboration. Ann Intern Med.

[31] Wengenmayer T, Duerschmied D, Graf E, Chiabudini M, Benk C, Mühlschlegel S, Philipp A, Lubnow M, Bode C, Staudacher DL (2019). Development and validation of a prognostic model for survival in patients treated with venoarterial extracorporeal membrane oxygenation: the PREDICT VA-ECMO score. Eur Heart J Acute Cardiovasc Care.

[32] Choi KH, Yang JH, Park TK, Lee JM, Song YB, Hahn J-Y, Choi S-H, Choi J-H, Cho YH, Sung K (2019). Risk prediction model of in-hospital mortality in patients with myocardial infarction treated with venoarterial extracorporeal membrane oxygenation. Rev Esp Cardiol (Engl Ed.

[33] Hofmann B, Gmelin MJ, Metz D, Raspé C, Wienke A, Treede H, Simm A (2018). Cardiac surgery score (CASUS) improves outcome prediction in patients treated with extracorporal life support (ECLS). Perfusion.

[34] Worku B, Khin S, Gaudino M, Avgerinos D, Gambardella I, D’Ayala M, Ramasubbu K, Gulkarov I, Salemi A (2019). A simple scoring system to predict survival after venoarterial extracorporeal membrane oxygenation. J Extra Corpor Technol.

[35] Park SB, Yang JH, Park TK, Cho YH, Sung K, Chung CR, Park CM, Jeon K, Song YB, Hahn J-Y (2014). Developing a risk prediction model for survival to discharge in cardiac arrest patients who undergo extracorporeal membrane oxygenation. Int J Cardiol.

[36] Burrell AJC, Pellegrino VA, Wolfe R, Wong WK, Cooper DJ, Kaye DM, Pilcher DV (2015). Long-term survival of adults with cardiogenic shock after venoarterial extracorporeal membrane oxygenation. J Crit Care.

[37] Wang L, Yang F, Wang X, Xie H, Fan E, Ogino M, Brodie D, Wang H, Hou X (2019). Predicting mortality in patients undergoing VA-ECMO after coronary artery bypass grafting: the REMEMBER score. Crit Care.

[38] Schmidt M, Burrell A, Roberts L, Bailey M, Sheldrake J, Rycus PT, Hodgson C, Scheinkestel C, Cooper DJ, Thiagarajan RR (2015). Predicting survival after ECMO for refractory cardiogenic shock: the survival after veno-arterial-ECMO (SAVE)-score. Eur Heart J.

[39] Chen W-C, Huang K-Y, Yao C-W, Wu C-F, Liang S-J, Li C-H, Tu C-Y, Chen H-J (2016). The modified SAVE score: predicting survival using urgent veno-arterial extracorporeal membrane oxygenation within 24 hours of arrival at the emergency department. Crit Care.

[40] Muller G, Flecher E, Lebreton G, Luyt C-E, Trouillet J-L, Bréchot N, Schmidt M, Mastroianni C, Chastre J, Leprince P (2016). The ENCOURAGE mortality risk score and analysis of long-term outcomes after VA-ECMO for acute myocardial infarction with cardiogenic shock. Intensive Care Med.

[41] Siao F-Y, Chiu C-W, Chiu C-C, Chang Y-J, Chen Y-C, Chen Y-L, Hsieh Y-K, Chou C-C, Yen H-H (2020). Can we predict patient outcome before extracorporeal membrane oxygenation for refractory cardiac arrest?. Scand J Trauma Resusc Emerg Med.

[42] Lee SW, Han KS, Park JS, Lee JS, Kim SJ (2017). Prognostic indicators of survival and survival prediction model following extracorporeal cardiopulmonary resuscitation in patients with sudden refractory cardiac arrest. Ann Intensive Care.

[43] Peigh G, Cavarocchi N, Keith SW, Hirose H (2015). Simple new risk score model for adult cardiac extracorporeal membrane oxygenation: simple cardiac ECMO score. J Surg Res.

[44] Becher PM, Twerenbold R, Schrage B, Schmack B, Sinning CR, Fluschnik N, Schwarzl M, Waldeyer C, Seiffert M, Clemmensen P (2020). Risk prediction of in-hospital mortality in patients with venoarterial extracorporeal membrane oxygenation for cardiopulmonary support: the ECMO-ACCEPTS score. J Crit Care.

[45] Ayers B, Wood K, Gosev I, Prasad S (2020). Predicting survival after extracorporeal membrane oxygenation by using machine learning. Ann Thorac Surg.

[46] Tongyoo S, Chanthawatthanarak S, Permpikul C, Ratanarat R, Promsin P, Kongsayreepong S (2022). Extracorporeal membrane oxygenation (ECMO) support for acute hypoxemic respiratory failure patients: outcomes and predictive factors. J Thorac Dis.

[47] Schmidt M, Bailey M, Sheldrake J, Hodgson C, Aubron C, Rycus PT, Scheinkestel C, Cooper DJ, Brodie D, Pellegrino V (2014). Predicting survival after extracorporeal membrane oxygenation for severe acute respiratory failure. The respiratory extracorporeal membrane oxygenation survival prediction (RESP) score. Am J Respir Crit Care Med.

[48] Baek MS, Chung CR, Kim HJ, Cho WH, Cho Y-J, Park S, Park SY, Kang BJ, Kim J-H, Park SH (2018). Age is major factor for predicting survival in patients with acute respiratory failure on extracorporeal membrane oxygenation: a Korean multicenter study. J Thorac Dis.

[49] Cheng Y-T, Wu M-Y, Chang Y-S, Huang C-C, Lin P-J (2016). Developing a simple preinterventional score to predict hospital mortality in adult venovenous extracorporeal membrane oxygenation: a pilot study. Medicine.

[50] Roch A, Hraiech S, Masson E, Grisoli D, Forel J-M, Boucekine M, Morera P, Guervilly C, Adda M, Dizier S (2014). Outcome of acute respiratory distress syndrome patients treated with extracorporeal membrane oxygenation and brought to a referral center. Intensive Care Med.

[51] Pappalardo F, Pieri M, Greco T, Patroniti N, Pesenti A, Arcadipane A, Ranieri VM, Gattinoni L, Landoni G, Holzgraefe B (2013). Predicting mortality risk in patients undergoing venovenous ECMO for ARDS due to influenza A (H1N1) pneumonia: the ECMOnet score. Intensive Care Med.

[52] Enger T, Philipp A, Videm V, Lubnow M, Wahba A, Fischer M, Schmid C, Bein T, Müller T (2014). Prediction of mortality in adult patients with severe acute lung failure receiving veno-venous extracorporeal membrane oxygenation: a prospective observational study. Crit Care.

[53] Schmidt M, Zogheib E, Rozé H, Repesse X, Lebreton G, Luyt C-E, Trouillet J-L, Bréchot N, Nieszkowska A, Dupont H (2013). The PRESERVE mortality risk score and analysis of long-term outcomes after extracorporeal membrane oxygenation for severe acute respiratory distress syndrome. Intensive Care Med.

[54] Montero S, Slutsky AS, Schmidt M (2018). The PRESET-Score: the extrapulmonary predictive survival model for extracorporeal membrane oxygenation in severe acute respiratory distress syndrome. J Thorac Dis.

[55] Müller T, Schroll S, Philipp A, Karagiannidis C, Amann M, Lunz D, Langgartner J, Bein T, Fischer M, Lubnow M (2013). The ECMOnet score: a useful tool not to be taken absolutely. Intensive Care Med.

[56] Brunet J, Valette X, Buklas D, Lehoux P, Verrier P, Sauneuf B, Ivascau C, Dalibert Y, Seguin A, Terzi N (2017). Predicting survival after extracorporeal membrane oxygenation for ARDS: an external validation of RESP and PRESERVE scores. Respir Care.

[57] Shouval R, Bondi O, Mishan H, Shimoni A, Unger R, Nagler A (2014). Application of machine learning algorithms for clinical predictive modeling: a data-mining approach in SCT. Bone Marrow Transplant.

[58] Sandri M, Berchialla P, Baldi I, Gregori D, De Blasi RA (2014). Dynamic Bayesian networks to predict sequences of organ failures in patients admitted to ICU. J Biomed Inform.

[59] Van Calster B, McLernon DJ, van Smeden M, Wynants L, Steyerberg EW, Bossuyt P, Collins GS, Macaskill P, McLernon DJ, Moons KGM (2019). Calibration: the Achilles heel of predictive analytics. BMC Medicine.

[60] Keuning BE, Kaufmann T, Wiersema R, Granholm A, Pettilä V, Møller MH, Christiansen CF, Castela Forte J, Snieder H, Keus F (2020). Mortality prediction models in the adult critically ill: a scoping review. Acta Anaesthesiol Scand.

[61] Vincent J-L, Moreno R (2010). Clinical review: scoring systems in the critically ill. Crit Care.

[62] Marshall IJ, Wallace BC (2019). Toward systematic review automation: a practical guide to using machine learning tools in research synthesis. Syst Rev.

