# Supplementary material for: Predictive models in extracorporeal membrane oxygenation (ECMO): a systematic review
Source: Syst Rev. 2023 Mar 15;12:44. doi: 10.1186/s13643-023-02211-7 (PMC10015918; doi:10.1186/s13643-023-02211-7)
Supplement: Supplementary file 1 — Additional file 1: Table S1. Search string created by Polyglot Research tool. Table S2. Details of ECMO scores. [file 13643_2023_2211_MOESM1_ESM.docx]

***TABLES***

*Table S1. Search string created by Polyglot Research tool.*

| **Data source** | **String** |
| --- | --- |
| Medline (Ovid - <https://ovidsp.dc1.ovid.com/ovid-b/ovidweb.cgi>) | “exp "extracorporeal membrane oxygenation"/ OR (extracorporeal.af. AND membrane.af. AND oxygenation.af.) OR "extracorporeal membrane oxygenation".af. OR ecmo.af.”, |
| Embase (Ovid - <https://ovidsp.dc1.ovid.com/ovid-b/ovidweb.cgi>) | “exp "extracorporeal membrane oxygenation"/ OR (extracorporeal.af. AND membrane.af. AND oxygenation.af.) OR "extracorporeal membrane oxygenation".af. OR ecmo.af.”, |
| CINAHL (Ebsco - <https://web.s.ebscohost.com/ehost/search/advanced?vid=1&sid=5136d966-8f44-4d48-a42d-14e50aa4dee7%40redis>) | “(MH "extracorporeal membrane oxygenation+") OR (extracorporeal AND membrane AND oxygenation) OR "extracorporeal membrane oxygenation" OR ecmo” and Scopus “"extracorporeal membrane oxygenation" OR (extracorporeal AND membrane AND oxygenation) OR "extracorporeal membrane oxygenation" OR ecmo” |
| Scopus (Scopus – <https://www.scopus.com/search/form.uri?display=advanced>) | INDEXTERMS("extracorporeal membrane oxygenation") OR (ALL(extracorporeal) AND ALL(membrane) AND ALL(oxygenation)) OR ALL("extracorporeal membrane oxygenation") OR ALL(ecmo) |

*Table S2: Details of ECMO scores.*

| **Score Name** | **First author, year** | **Year** | **Country** | **Sample Size** | **Items required** | **ECMO type** | **Type of validation** |
| --- | --- | --- | --- | --- | --- | --- | --- |
| PREDICT VA-ECMO score[31] | Wengenmayer | 2018 | Germany - Europe | 449 | 1. Lactate (mmol/l) 2. pH 3. standard bicarbonate concentration (mmol/l) | VA-ECMO | External |
| AMI-ECMO score[32] | Choi | 2019 | South Korea - Asia | 145 | 1. Age (years) 2. Body mass index (kg m–2) 3. Glasgow coma score 4. Lactate (mmol/l) 5. Culprit lesion location 6. Successful revascularization | VA-ECMO | Internal |
| CASUS score[33] | Hoffmann | 2017 | Germany –  Europe | 90 | 1. Age (years) 2. Urinary output 3. Lactate (mmol/l) 4. Activated partial thromboplastin time | VA-ECMO | Internal |
| Worku et al. score[34] | Worku | 2019 | USA - America | 100 | 1. Lactate (mmol/l) 2. Platelet concentration (×1000 μl–1) 3. Albumin (g/dl) 4. Age (years) 5. Intubated (days) | VA-ECMO | Internal |
| New ECPR score[35] | Park | 2014 | South Korea - Asia | 152 | 1. Age (years) 2. Diabetes 3. Cardiomyopathy 4. cardiopulmonary resuscitation duration (minutes) 5. Pulse pressure (mmHg) 6. Mean arterial pressure (mmHg) 7. SOFA score 8. Pulseless electrical activity 9. Asystole 10. Ventricular Fibrillatoion / Pulseless Ventricular Tachycardia | VA-ECMO | Internal |
| LOVE score[36] | Burrell |  | Australia - Oceania | 125 | 1. Cardiomyopathy 2. Bilirubin concentration (mg dl–1) 3. renal replacement therapy 4. Lactate (mmol/l) | VA-ECMO | Internal |
| REMEMBER score[37] | Wang | 2019 | China - Asia | 106 | 1. Age (years) 2. Left main disease 3. Inotropic score 4. Creatine Kinase-MB IU/l 5. Creatinine 6. Platelet concentration (×1000 μl–1) | VA-ECMO | Internal |
| SAVE score[38] | Schmidt | 2015 | Australia - Oceania | 4007 | 1. Myocarditis 2. Refractory ventricular ventricular tachycardia/ventricular fibrillation 3. Post heart or lung transplantation 4. Congenital heart disease 5. Age (years) 6. Weight (kg) 7. Liver failure 8. central nervous system dysfunction (%) 9. Renal failure 10. Chronic renal failure 11. Duration of intubation prior to ECMO (h) 12. Peak inspiratory pressure 13. Pre‑ECMO cardiac arrest 14. Diastolic pressure 15. Pulse pressure (mmHg) 16. Bicarbonate (mmol/l) | VA-ECMO | External |
| Modified SAVE score[39] | Chen | 2016 | China | 154 | 1. SAVE Score 2. Lactate (mmol/l) | VA-ECMO | Internal |
| ENCOURAGE score[40] | Muller | 2016 | France - Europe | 138 | 1. Age (years) 2. Sex, male (%) 3. Body mass index (kg m–2) 4. Glasgow coma score 5. Creatinine 6. Lactate (mmol/l) 7. Prothrombin activity (%) | VA-ECMO | Internal |
| Siao et al. score[41] | Siao | 2020 | Taiwan - America | 112 | 1. Extracorporeal cardiopulmonary resuscitation 2. cardiopulmonary resuscitation duration (minutes) 3. Heart rate (bpm) | Both types of ECMO | Internal |
| Lee et al. score[42] | Lee | 2017 | Korea - Asia | 111 | 1. Age (years) 2. Cardiopulmonary resuscitation duration (minutes) 3. Ventricular Fibrillatoion / Pulseless Ventricular Tachycardia 4. Pulseless electrical activity 5. Return of spontaneous circulation before ECMO pump on | Both ECMO types | Internal |
| Simple cardiac score[43] | Peigh | 2015 | Pennsylvania - America | 73 | 1. RIFLE score 2. Lactate (mmol/L) 3. Postcardiotomy shock (%) | Both ECMO types | Internal |
| ECMO-ACCEPTS score[44] | Becher | 2019 | Germany | 8351 | 1. Age (years) 2. Urgent admission 3. Extracorporeal cardiopulmonary resuscitation 4. atrial fibrillation 5. coronary artery disease 6. Chronic heart failure of other causes 7. primary pulmonary hypertension 8. hypertension 9. Post-ECMO Heart transplantation 10. acute coronary syndrome | VA-ECMO | Internal |
| DNN Score[45] | Ayers | 2020 | America | 282 | 1. Lactate (mmol/L) 2. Age (years) 3. Bilirubin concentration (mg dl–1) 4. Creatinine 5. Alanine aminotransferase (unit/L) 6. Phosphorus 7. Aspartate aminotransferase (unit/L) 8. Platelet concentration (×1000 μl–1) 9. pH 10. Alkaline phosphate 11. Potassium 12. Hematocrit (%) 13. Sodium 14. White blood cell count (K/uL) 15. Postcardiotomy shock (%) 16. Haemoglobin concentration (g dl–1) 17. Magnesium 18. Pre‑ECMO cardiac arrest 19. Sex, male (%) | VA-ECMO | Internal |
| SHOP score[54] | Tongyoo | 2022 | Tailandia | 65 | 1. SOFA score 2. Pre-ECMO hospital stay (days) 3. PaO2/FiO2 (mmHg) 4. pH | Both ECMO types | Internal |
| RESP score[46] | Schmidt | 2014 | France - Europe | 2495 | 1. Age (years) 2. Immunocompromised (%) 3. Pre‑ECMO mechanical ventilation days 4. Diagnosis leading to ECMO 5. central nervous system dysfunction (%) 6. Acute nonpulmonary-associated infection 7. Neuromuscular blockade agents 8. Inhaled nitric oxide 9. Bicarbonate infusion (%) 10. Pre‑ECMO cardiac arrest 11. PaCO2 (mmHg) 12. Peak inspiratory pressure | VV-ECMO | External |
| Modified RESP score[47] | Baek | 2018 | Korea – Asia | 209 | 1. Age (years) 2. RESP Score | VV-ECMO | Internal |
| PRESET score[23] | Hilder | 2017 | Germany - Europe | 141 | 1. Pre-ECMO hospital stay (days) 2. Lactate (mmol/l) 3. Mean arterial pressure (mmHg) 4. pH 5. Platelet concentration (×1000 μl–1) | VV-ECMO | External |
| Cheng et al. score[50] | Cheng | 2016 | Taiwan - America | 116 | 1. Pre‑ECMO mechanical ventilation days 2. Hematocrit (%) 3. SOFA score 4. Hospital stay (days) | VV-ECMO | Internal |
| Roch et al. score[51] | Roch | 2013 | France - Europe | 85 | 1. SOFA score 2. Age (years) 3. Pneumonia | VV-ECMO | Internal |
| ECMOnet score[48] | Pappalardo | 2012 | Italy – Europe | 134 | 1. Pre-ECMO hospital stay (days) 2. Bilirubin concentration (mg dl–1) 3. Creatinine 4. Haematocrit (%) 5. Mean arterial pressure (mmHg) | VV-ECMO | External |
| Model 1 (Enger) score[52] | Enger | 2014 | Germany - Europe | 304 | 1. Age (years) 2. Immunocompromised (%) 3. Minute ventilation (l/minute) 4. Haemoglobin concentration (g dl–1) 5. Lactate (mmol/l) | VV-ECMO | Internal |
| Model 2 (Enger) [52] | Enger | 2014 | Germany - Europe | 241 | 1. Age (years) 2. Immunocompromised (%) 3. Minute ventilation (l/minute) 4. Haemoglobin concentration (g dl–1) 5. Day 1 FiO2 (per 10%) 6. Day 1 fibrinogen (mg/dl) 7. Day 1 norepinephrine (μg/minute/10 kg) 8. Day 1 C-reactive protein (mg/L) | VV-ECMO | Internal |
| PRESERVE score[53] | Schmidt | 2013 | France - Europe | 140 | 1. Age (years) 2. Body mass index (kg m–2) 3. Immunocompromised (%) 4. SAPS II 5. Pre‑ECMO mechanical ventilation days 6. Prone positioning (%) 7. Positive end-expiratory pressure 8. Plateau airway pressure (cmH2O) | VV-ECMO | External |
